# Therapeutic Effect of Melatonin on CCl_4_-Induced Fibrotic Liver Model by Modulating Oxidative Stress, Inflammation, and TGF-β1 Signaling Pathway in Pinealectomized Rats

**DOI:** 10.1007/s10753-024-02101-7

**Published:** 2024-07-15

**Authors:** Derya Cinar, Eyup Altinoz, Hulya Elbe, Yasemin Bicer, Dilan Cetinavci, Ipek Ozturk, Tuncay Colak

**Affiliations:** 1 Department of Anatomy, School of Health Science, Karabuk University, Karabuk, Turkey; 2 Department of Medical Biochemistry, Faculty of Medicine, Karabuk University, Karabuk, Turkey; 3 Department of Histology and Embryology, Faculty of Medicine, Mugla Sıtkı Kocman University, Mugla, Turkey; 4 Department of Histology and Embryology, Mugla Training and Research Hospital, Mugla, Turkey; 5 Department of Anatomy, Faculty of Medicine, Kocaeli University, Kocaeli, Turkey

**Keywords:** liver fibrosis, inflammation, oxidative stress, CCl_4_, pinealectomy, melatonin

## Abstract

The study aimed to determine the CCl_4_-induced liver fibrosis model in pinealectomized rats and biochemically, immunohistochemically, and histopathologically investigate the therapeutic effect of melatonin on liver fibrosis. The surgical procedure for pinealectomy was performed at the beginning of the study, and the sham and pinealectomized rats were administered CCl_4_ dissolved in corn oil (1:1) alone every other day to induce liver fibrosis or together with melatonin (10 mg/kg) therapy for 15 days. Melatonin is an essential therapeutic agent and offers an alternative therapeutic strategy in CCl_4_-induced liver fibrosis by suppressing inflammation, oxidative stress, and the TGF-β1 signaling pathway. Treatment with melatonin ameliorated CCl_4_-induced liver fibrosis by restoring hepatocellular damage and reducing plasma AST, ALT, and ALP values. Melatonin increases the activity of SOD and CAT, which are important enzymes for antioxidant defence, and raises GSH levels, which further enhances antioxidant function. Also, melatonin reduced hepatic inflammation (IL-6 and IL-1β) and oxidative stress indices. Moreover, histopathological changes and immunohistochemical expression of TGF-β1 were restored following melatonin supplementation in the CCl_4_-induced liver fibrosis model in pinealectomized rats. Our study shows that melatonin supplementation has a beneficial effect in protecting the liver fibrosis induced by CCl_4_ in pinealectomized rats.

## INTRODUCTION

The liver acts like an exocrine gland due to its function to discharge bile into the duodenum via the bile duct and like an endocrine gland with significant metabolic functions due to its function to synthesise and release proteins such as glucose, glycoproteins, globulins, fibrinogen, albumin, and prothrombin to the blood [1]. The liver minimises the harmful effects of external compounds. The liver, also involved in drug and xenobiotic metabolisms, could be damaged by detoxification products [2]. According to the World Health Organization (WHO) data, 2.4 million people annually die due to liver diseases. It is a global health problem with a high prevalence in developing countries, and liver diseases affect hundreds of millions of people [3].

Carbon tetrachloride (CCl_4_), a known pre-oxidant, is the most common xenobiotic to develop experimental liver injury models. CCI_4_ is a colourless, transparent, volatile liquid. Free radicals produced by CCl_4_ metabolism lead to damage in the organ [4]. The cytochrome P-450 enzyme system in the liver converts CCl4 into intermediate metabolite trichloromethyl (^.^CCl_3_), a reactive toxic substance, and leads to the formation of trichloromethylperoxy (^.^OOCCl_3_) when oxygen is present. Lipid peroxidation starts after the trichloromethylperoxine reaction with polyunsaturated fatty acids, or it destroys the cellular membrane by covalent binding to fats and proteins, thus causing liver. [5]. Liver damage activates Kupffer cells, leading to ROS production, especially peroxy-nitrites. It leads to the secretion of early inflammation mediators such as superoxide anions that, in turn, produce hydrogen peroxides (H_2_O_2_), resulting in oxidative stress. A balance exists between oxidants and antioxidants. [6] If abnormal levels of oxidants are produced or if antioxidants do their job less than they should, in other words, if there are disturbances between oxidant and antioxidant levels, harmful effects of oxidant molecules are observed on proteins, carbohydrates, and nucleic acids [7]. Thus, equilibrium between free radical activity and the antioxidant defence system is a significant requirement to prevent oxidative stress-induced cellular damage [8]. Antioxidants have a vital role to play when it comes to preventing liver damage caused by CCl_4_.

One of the primary functions of the pineal gland is melatonin secretion. Melatonin (MLT) is released at night and regulates the circadian cycle [9]. MLT could prevent free radical damage by neutralizing them [10]. MLT is an indoleamine derived from tryptophan, which, along with serotonin, is an essential amino acid [11]. MLT could directly scavenge free radicals and is a powerful antioxidant which reduces oxidative stress by increasing other antioxidant enzyme activities [12]. In short, the direct effect of melatonin is the reduction of free radical production due to its free radical scavenging effect, and its indirect effect includes the increase of the effect of other antioxidant enzymes [13].

We hypothesised that the antioxidant properties of melatonin could reduce the liver damage caused by CCl_4_. During our investigation, we examined the levels of malondialdehyde (MDA) as a lipid peroxidation marker end product, as well as total antioxidant status (TAS), total oxidant status (TOS), and oxidative stress index (OSI) levels, which are all markers of oxidative stress. We also investigated liver superoxide dismutase (SOD) and catalase (CAT) activities and glutathione (GSH) levels, as well as the serum aspartate aminotransferase (AST), alanine aminotransferase (ALT) and alkaline phosphatase (ALP), which are liver enzymes. We covered the levels of inflammatory cytokines, including interleukin-6 (IL-6) and interleukin-1 beta (IL-1β), in liver samples and performed histopathological examinations and immunohistochemical staining with TGF-β1.

The present study aimed to investigate the development and progression of the liver fibrosis model using CCl_4_ following melatonin deprivation by pinealectomy and the effect of exogenous melatonin supplementation on fibrotic liver tissue and to assess its potential therapeutic benefits.

## MATERIAL AND METHODS

### Experimental Animal

The study data were collected from 56 male Wistar albino rats weighing 250–300 g. The rats were obtained from Zonguldak Bulent Ecevit University's Experimental Animal Breeding and Research Center (ZBEÜN-DEHAM). The study was approved by the Zonguldak Bülent Ecevit University Animal Research Ethics Committee (Protocol No: 2021–17-01/07). The rats were divided into seven groups. (n:8). The rats were maintained at a constant temperature of 21 ± 1 C and exposed to 12 h of light and 12 h of darkness in a temperature-controlled room. The rats were fed with standard pellet chow and tap water ad libitum throughout the study. The cages were cleaned every other day, and the drinking water was changed daily. The experimental procedures were carried out in compliance with the guidelines set by the Animal Ethics Committee.

### Experimental Design

At the beginning of the study, the rats were randomly divided into seven groups, each consisting of eight animals, as follows;

- *Control:* Melatonin solution (max 0.5% ethyl alcohol) was administered to the control group rats daily for 15 days intraperitoneally (i.p).
- *Sham:* Rats in this group underwent sham surgery where only the scalp was incised and stitched without pinealectomy. They were also administered 0.5 ml/kg of corn oil via i.p every other day.
- *Sham* + *CCl*_*4*_*:* Rats in this group underwent sham surgery, and CCI_4_ dissolved in corn oil (1:1, 1 ml/kg bw) was administered via i.p to the rats for 15 days every other day (a total of eight injections) [14, 15].
- *Pinealectomy (PINX):* The rats were subjected to a PINX surgery procedure and administered 0.5 ml/kg of corn oil via i.p injection every other day for 15 days.
- *PINX* + *CCl4:* The rats underwent PINX surgery, and CCI4 dissolved in corn oil (1:1, 1 ml/kg bw) was administered via i.p for 15 days every other day.
- *PINX* + *MLT:* The rats underwent PINX surgery, and 10 mg/kg melatonin solution (max 0.5% ethyl alcohol) was administered via i.p for 15 days [16].
- *PINX* + *CCl*_*4*_ + *MLT:* Rats were treated with 10 mg/kg melatonin solution via i.p for 15 days, along with PINX + CCl4 applications.

All applications were applied to rats simultaneously in a volume of 1 ml/kg bw. On the 16th day, blood and liver tissues were collected after the rats were decapitated under xylasin/ketamine anaesthesia.

### Pinealectomy Surgery

The rats were given anaesthesia by administering 8 mg/kg of xylazine and 80 mg/kg of ketamine through intraperitoneal injection. The rat was secured to the stereotaxic frame, and its scalp was cut to expose the exact lambda point between its eyes. The periosteum was stripped back and prepared for the study. A circular incision with a diameter of 3 mm was conducted with a Proxxon MICROMOT 50/E German micro milling drill on this section of the scalp and the whole gl. pinealis, located at the bottom of the venous sinus, was removed without disintegration. The procedures were conducted as in our previous study [17].

### Tissue and Blood Sample Collection

In the last stage of the experiment, blood was collected from the abdominal veins of the anaesthetised rats into tubes, and then the rats were decapitated. Liver tissues were collected using sterile methods and washed with physiological saline to remove the excess blood. The liver tissue was then separated into two sections. One of the sections was placed in 10% neutral buffer formalin for histological analysis, and the other was wrapped in aluminium foil for biochemical analysis and stored in the deep freezer at -80 Cº until the analysis day. The blood samples were left at room temperature for one hour to avoid clotting. Afterwards, they were centrifuged at 4000 revolutions per minute for 10 min to obtain serum. The serum samples were analyzed to determine liver function tests.

### Biochemical Analysis

Liver tissue samples were removed from the deep freezer and weighed on the analysis day. To create 10% homogenates, we added phosphate buffer with a pH of 7.5. The samples were homogenized with an automatic homogenizer (Hangzhou Bioprep-24) at 10,000 rpm for 3 min. Supernatants were obtained by centrifuging liver tissue homogenates at 5000 rpm for 30 min at + 4 ºC before determining MDA levels. The levels of CAT, SOD, GSH, protein, TAS, TOS, OSI, IL-6 and IL-1β were measured in the supernatants.

#### Oxidative Stress Indices

The MDA content of the homogenates was evaluated using the method proposed by Ohkawa et al. [18]. The homogenates were mixed with 1% phosphoric acid (H3PO4) and 6% thiobarbituric acid in lidded glass tubes. The samples were subjected to a 45-min boiling water bath and then extracted using n-butanol. Then, MDA levels were determined by measuring the pink colour using an ELISA reader at a wavelength of 535 nm. The MDA results were expressed as nanomoles per gram of wet tissue.

The GSH content of the supernatant was analyzed using the method developed by Ellman [19]. Following the elimination of the proteins, the samples could react with 5,5'-dithiobis 2-nitrobenzoic acid and transform into a yellow-greenish colour. GSH level was measured with the ELISA device at 410 nm. The GSH findings were expressed as nanomoles per gram of wet tissue.

The SOD activity was calculated using the method proposed by Sun [20]. The method allows the generation of superoxide radicals in the test environment due to xanthine-xanthine oxidase reaction. Blue formazan was formed with the reduction of NBT (nitro blue tetrazolium) by the radicals, the formazan was separated based on the study group, and SOD activity was determined with the ELISA reader at 560 nm. SOD activity was expressed as U/g.

The CAT activity was calculated using the method proposed by Aebi [21]. Phosphate buffer with H2O2 was mixed with the supernatant, and the breakdown of H_2_O_2_ into H_2_O and O2 caused the absorbance change at 240 nm. The CAT activity was determined by measuring the change in absorbance. CAT activity was expressed as K/g protein.

Protein concentration was determined using Lowry’s method [22]. The protein content of the blue-coloured product was recorded with blind measurement and an ELISA reader at 540nm. Protein levels were used to calculate SOD and CAT activities.

TOS was analyzed using Erel's method [23]. The tissue TOS level was determined using the Total Oxidant Status kit (Rel Assay Diagnostics, Gaziantep, Turkey). Tissue TOS content was measured at 530 nm using an ELISA reader and expressed as µmol H_2_O_2_ equiv /L.

TAS was analyzed using Erel's method [24]. Tissue TAS content was determined using the Total Antioxidant Status kit (Rel Assay Diagnostics, Gaziantep, Turkey). The antioxidant molecules' decolourisation was measured by conducting some measurements. Reagent 1 was added to the supernatants in microplates, followed by the first reading at 660 nm. Then, another reading was performed after adding Reagent 2. TAS was calculated based on the difference between the two readings. Trolox, a water-soluble vitamin E equivalent, was used for calibration. TAS content was expressed as mmol Trolox Equiv/L.

The OSI of liver tissue was determined using Erel's method [25]. To calculate OSI, TOS was divided by TAS. The formula used was OSI (Arbitrary Unit) = TOS (μmol H_2_O_2_ eqv/1) / [(TAS (mmol Trolox eqv/l) × 10]. The results are presented in arbitrary units (AU).

#### Liver Function Tests

Serum AST, ALT, and ALP levels were determined using analysis kits (Abbott, Abbott Park. Illinois, USA) and an autoanalyser (Architect C8000). The findings are presented as mg/dl.

#### Inflammatory Indices

IL-1β and IL-6 levels in liver tissue were determined using the BT LAB rat ELISA kits (Cat no: E0119Ra and Cat no: E0135Ra, respectively). The results are presented as ng/ml.

### Histopathological Analysis

The liver tissue pieces were fixed in 10% neutral formalin buffer (NTF). One day later, the liver tissues were incised into 3-4 mm sections; the sections were placed in cartridges and fixed in NTF for 24 hours. Then, the sections were washed under tap water for 24 hours. The tissues were trimmed and passed through a series of alcohol solutions with increasing concentrations (70%, 80%, 95% and 96%) for 2x45 minutes. They were cleaned with xylol for 2x30 minutes. They were then embedded in paraffin for 2x30 minutes. A Leica TP 1020 device was employed to monitor the tissues. After embedding was completed, the paraffin blocks were left to cool. Sections of 5μm were cut from paraffin blocks using a Leica FINESSE ME+ microtome onto gelatin-coated slides for light microscopic and immunohistochemical examinations. The slides were placed in an oven at 37°C for 2 hours to increase the tissue adhesion on the slide. The tissue sections were treated with hematoxylin-eosin staining to examine their overall histological appearance. Harmful factors were determined using the semi-quantitative method in the liver based on the degree and frequency of histopathological differences. Sections were observed based on ten contexts for congestion, sinusoidal dilatation, and inflammatory cell infiltration. They were scored as 0 (no change), 1 (mild), 2, and 3 (severe) based on the damage rate. Nikon Eclipse 80i light microscope and Nikon image analysis system were employed to photograph and score the sections [26].

### Immunohistochemical (IHC) Analysis

The sections were put in slides coated with polylysine for IHC evaluation. The slides were rehydrated and then put in citrate buffer with a pH of 7.6 and heated for 20 min in a microwave oven. The slides were allowed to cool for just 20 min at room temperature and then washed with phosphate-buffered saline (PBS). Next, they were incubated in 0.3% H_2_O_2_ solution for 7 min before being washed in PBS. The slides were then exposed to anti-TGFβ1 (Bt Lab, Cat no: BT-Apo8976) for 60 min. They were washed with PBS and incubated with biotinylated goat antipolyvalent and streptavidin peroxidase (SHP 125, ScyTek Laboratories, USA) for 10 min at room temperature. Then, the slides were stained with chromogen + substrate for 15 min. They were then counterstained with Mayer's hematoxylin (M06002, Bio-Optica, ITA) for 1 min, washed with water, and dehydrated. The antibody was used following the manufacturer's instructions, which resulted in a brown tint indicating Anti-TGFβ1 immunostaining. The severity of the staining was graded on a scale of 0 to 3 based on its intensity: absence (0), mild (1), moderate (2), and severe (3). The sections were evaluated using a Nikon Eclipse 80i light microscope and Nikon Image Analysis system, and the H-score was determined. The immunohistochemical staining score was determined for each section based on the H-Score method (I x PC) (I: degree of staining, PC: percentage of cells stained at each degree).

### Statistical Analysis

The IBM SPSS version 14.0 for Windows was utilized to conduct statistical analyses. All the results were expressed as arithmetic ± standard error (SE). It was determined that the measurable differences between the study groups were not distributed normally based on the Shapiro–Wilk normality test results (p > 0.05). Non-parametric statistical analysis was used to compare study groups based on all variables. Kruskal–Wallis variance analysis was used, and then the Mann–Whitney U-test was applied for pairwise comparisons. If the obtained p-value is less than 0.05, it is considered statistically significant.

## RESULTS

### Exogenous Melatonin Supplementation Effects on Oxidative Stress and Antioxidant Parameters in CCl_4_-Induced Liver Fibrosis Model

Several studies have demonstrated that exposure to CCl4 results in the overproduction of ROS and disrupts the antioxidant balance of the liver tissue, ultimately leading to liver fibrosis [27, 28]. To evaluate the hepatic oxidative stress induced by CCl4 after pinealectomy surgery, as well as the protective potential of MLT, we measured both enzymatic (SOD and CAT) and non-enzymatic (MDA, GSH, TAS, TOS, and OSI) parameters. Oxidant/antioxidant parameters of liver tissue are presented in Tables 1 and 2. According to our research, a surgical procedure called pinealectomy (PINX) results in a significant increase in MDA (*p* < 0.001), TOS (*p* < 0.05), and OSI (*p* < 0.001) indices compared to the control and sham. The PINX procedure also causes a depletion in GSH (*p* < 0.05) and TAS (*p* < 0.001) levels and a reduction in CAT and SOD activity (*p* < 0.001) due to melatonin deprivation, which results in a deterioration in the antioxidant balance of the liver tissue. Compared to the control and sham groups, as well as the group that underwent pinealectomy surgery (PINX), the administration of CCl_4_ resulted in a significant increase in liver MDA levels (*p* < 0.001) and a remarkable decrease in GSH levels (*p* < 0.001). Furthermore, the effect of post-pinealectomy CCl_4_ administration (PINX + CCl_4_) on the animals' liver MDA and GSH levels was almost identical to that of the CCl_4_ administration. After inducing an experimental liver fibrosis model using CCl_4_, significant reductions in SOD and CAT activities (*p* < 0.001) were observed in comparison to the control and sham groups, as well as the PINX groups. The administration of CCl_4_ after post-pinealectomy (PINX + CCl_4_) showed similar effects compared to the aforementioned groups. Meanwhile, significant changes (*p* < 0.001) were observed in the levels of the tissue TAS, TOS, and OSI, which were directly proportional to the extent of liver damage caused by CCl_4_ (Table 2). Also, post-pinealectomy CCl_4_ administration (PINX + CCl_4_) led to changes in TAS (*p* < 0.005), TOS (*p* < 0.01), and OSI (*p* < 0.001) relative to CCl_4_ administration without pinealectomy (Sham + CCl_4_). However, co-administration of CCl_4_ with MLT following pinealectomy (PINX + CCl_4_ + MLT) gave rise to a significant recovery in depleted GSH content (*p* < 0.001) while showing a notable decrease in elevated MDA levels (*p* < 0.001) in comparison to the Sham + CCl_4_ and PINX + CCl_4_ groups. Obviously, co-administration of CCl_4_ with MLT following pinealectomy (PINX + CCl_4_ + MLT) supported the tissue antioxidant system and recovered both SOD (*p* < 0.005) and CAT (*p* < 0.001) activity as compared to Sham + CCl_4_ and PINX + CCl_4_ groups. Upon comparing to self-administration of CCl_4_ (Sham + CCl_4_ and PINX + CCl_4_ groups), it was observed that the recovery of oxidant + antioxidant disturbances using CCl_4_ was achieved through co-treatment of CCl_4_ with MLT following pinealectomy (PNX + CCl_4_ + MLT). This treatment resulted in the elevation of TAS levels (*p* < 0.001) and restoration of TOS and OSI levels (*p* < 0.001).

**Table 1:** Comparison of Average Tissue Oxidant-Antioxidant Parameters

Groups	MDA (nmol/gwt)	GSH (nmol/gwt)	SOD (U/g protein)	CAT (K/g protein)
Control	615.68 ± 18.77	2849.48 ± 184.40	42.01 ± 2.17	51.71 ± 1.23
Sham	623.62 ± 24.56	2684.66 ± 71.04	38.49 ± 3.19	52.76 ± 1.30
PINX	865.72 ± 51.34^a,b^	2113.93 ± 167.31^ g,h^	24.61 ± 1.19^a,b^	32.86 ± 1.72^a,b^
Sham + CCI_4_	1731.44 ± 102.69^a,b,c^	1056.96 ± 83.65^a,b,c^	15.33 ± 2.75^a,b,d^	16.40 ± 2.33^a,b,e^
PINX + CCl_4_	1803.38 ± 106.63^a,b,c^	851.96 ± 46.51^a,b,c^	13.22 ± 0.76^a,b,e^	9.02 ± 0.95^a,b,e,n^
PINX + MLT	595.71 ± 13.40^c,d,e^	2647.42 ± 115.17^d,e^	38.17 ± 2.28^e,f,h^	45.44 ± 1.50^ m,c,e,f,h^
PINX + CCl_4_ + MLT	890.76 ± 57.21^a,b,d,e,f^	1333.23 ± 61.11^a,b,c,e,f^	26.51 ± 2.00^a,c,g,h,k^	30.29 ± 1.00^a,b,f,h,p^

Data are expressed as the arithmetic mean ± SE (*n* = 8). MDA, malondialdehyde; GSH, reduced glutathione; SOD, superoxide dismutase; CAT, catalase; gwt, gram wet tissue. Superscripts represents the statistically significant difference

MDA, GSH, ^a^ *p* < 0.001 vs Control, ^b^ p < 0.001 vs Sham, ^c^ *p* < 0.001 vs PINX, ^d^ *p* < 0.001 vs Sham + CCl_4_, ^e^ *p* < 0.001 vs PINX + CCl_4_, ^f^ *p* < 0.001 vs PINX + MLT, ^g^*p* < 0.01 vs Control, ^h^ *p* < 0.05 vs Sham. SOD, CAT; ^a^ *p* < 0.001 vs Control, ^b^ *p* < 0.001 vs Sham, ^c^ *p* < 0.005 vs Sham, ^d^ *p* < 0.005 vs PINX, ^e^ *p* < 0.001 vs PINX, ^f^*p* < 0.001 vs Sham + CCl_4_, ^g^ *p* < 0.005 vs Sham + CCl_4_, ^h^ *p* < 0.001 vs PINX + CCl_4_, ^k^ *p* < 0.005 vs PINX + MLT, ^m^ *p* < 0.01 vs Control, ^n^ *p* < 0.05 vs Sham + CCl_4_, ^p^ *p* < 0.001 vs PINX + MLT

**Table 2:** Comparison of Mean TAS, TOS, and OSI Values in Liver Tissue

Groups	TAS (nmol Trolox Equiv/L)	TOS (µmol H_2_O_2_ Equiv/L)	OSI (Arbitrary Unit)
Control	1.84 ± 0.03	18.11 ± 0.64	990.01 ± 49.61
Sham	1.61 ± 0.02^a^	19.66 ± 0.72	1224.20 ± 51.77^ m^
PINX	1.22 ± 0.02^a,b^	20.36 ± 0.73^ m^	1666.74 ± 77.50^a,b^
Sham + CCI_4_	0.52 ± 0.03^a,b,d^	35.06 ± 2.31^a,b,d^	6792.00 ± 377.46^a,b,d^
PINX + CCl_4_	0.38 ± 0.02^a,b,d,e^	45.73 ± 1.58^a,b,d,p^	12028.65 ± 697.26^a,d,f^
PINX + MLT	1.79 ± 0.03^c,d,f,g^	18.61 ± 1.24^f,g^	1040.91 ± 74.16^d,f,g^
PINX + CCl_4_ + MLT	1.10 ± 0.01^a,b,d,f,g,h^	30.55 ± 2.00^b,n,g,h^	2744.77 ± 156.52^a,b,d,f,g,h^

Data are expressed as the arithmetic mean ± SE (*n* = 8). TAS, Total Antioksidant Status; TOS, Total Oksidant Status; OSI, Oxidative Stress İndex. Superscripts represents the statistically significant difference

^a^ *p* < 0.001 vs Control, ^b^ *p* < 0.001 vs Sham, ^c^ *p* < 0.005 vs Sham, ^d^ *p* < 0.001 vs PINX, ^e^ *p* < 0.005 vs Sham + CCl_4_, ^f^ *p* < 0.001 vs Sham + CCl_4_, ^g^ *p* < 0.001vs PINX + CCl_4_, ^h^ *p* < 0.001 vs PINX + MLT, ^m^ *p* < 0.05 vs Control, ^n^ *p* < 0.001 vs PINX, ^p^ *p* < 0.01vs Sham + CCl_4_

### Exogenous Melatonin Supplementation Effects on Liver Inflammation Markers in CCl_4_-Induced Liver Fibrosis Model

**Fig. 1:**
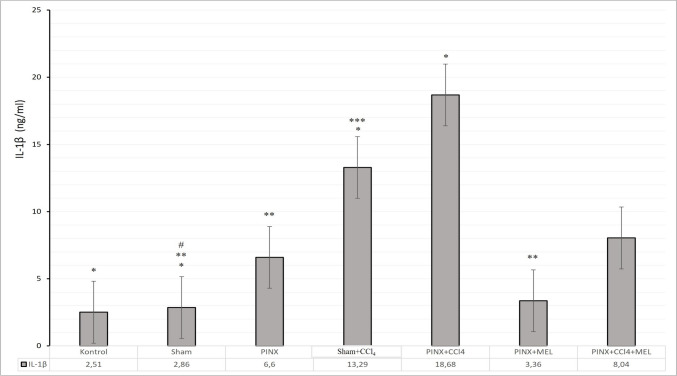
Effect of exogenous melatonin on IL-1β levels in CCl4-induced liver fibrosis.

**Fig. 2:**
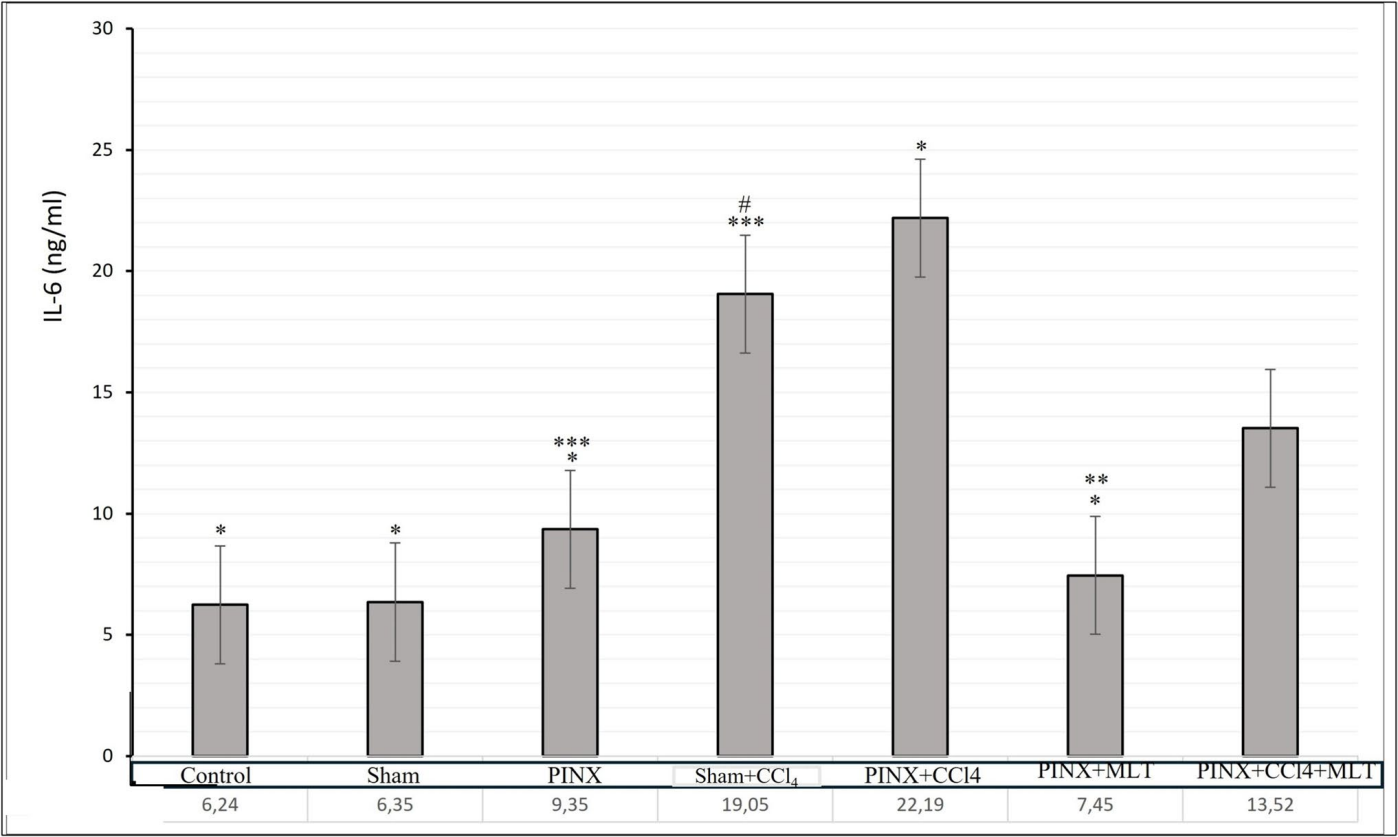
Effect of exogenous melatonin on IL-6 levels in CCl4-induced liver fibrosis.

CCl_4_ triggers the activation of Kupffer cells, resident macrophages in the liver, leading to large amounts of pro-inflammatory mediators and releasing some cytokines, including IL-6, by activating nuclear factor kappa (NF)-κB through phosphorylation. After the secretion of pro-fibrogenic cytokines, the activation process is inhibited, and these cytokines have the potential to induce hepatic fibrogenesis [29]. Thus, we decided to evaluate hepatic inflammation using CCl_4_ following pinealectomy surgery and the ameliorative effect of MLT by determining the levels of IL-1β and IL-6 in rat liver tissues. As shown in Fig. 1 and 2, pinealectomy-induced melatonin deprivation (PINX) led to significant increases in liver IL-1β (*p* < 0.005) and IL-6 (*p* < 0.001) levels relative to control and sham. In addition, self-administration of CCl_4_ (Sham + CCl_4_ and PINX + CCl_4_ groups) to induce liver fibrosis caused a significant increase in the liver levels of IL-1β and IL-6 (*p* < 0.001) compared to the control and sham. In contrast, co-treatment of CCl_4_ with MLT following pinealectomy (PNX + CCl_4_ + MLT) suppressed hepatic inflammation and resulted in a remarkable reduction of IL-1β and IL-6 levels (*p* < 0.05) in the fibrotic liver tissue upon comparing to Sham + CCl_4_ and PINX + CCl_4_.

### Exogenous Melatonin Supplementation Effects on Circulating Markers of Liver Function in CCl_4_-Induced Liver Fibrosis Model

The liver plays a vital role in breaking down drugs and other foreign substances, known as xenobiotics. Two enzymes, ALT and AST, are prominent in liver cells and are used to monitor the liver's health status in response to drugs and xenobiotics. The damage caused by CCl_4_ to the liver cells releases these enzymes into the bloodstream. On the other hand, increased activity of ALP indicates damage to the liver's bile ducts [28]. Therefore, we measured the serum AST, ALT, and ALP levels to monitor the liver's health status in the CCl_4_-induced liver fibrosis model. As presented in Table 3, pinealectomy surgery increased serum ALP levels, while there was no difference between serum ALT and AST levels of the control, sham, and PINX groups. Interestingly, self-administration of CCl_4_ (Sham + CCl_4_ and PINX + CCl_4_ groups) led to hepatocyte damage and increased serum ALT, AST, and ALP levels relative to the control, sham, and PINX groups (*p* < 0.001). A significant improvement of ALT (*p* < 0.005), AST (*p* < 0.05), and ALP (*p* < 0.001) levels was observed after co-treatment of CCl_4_ with MLT following pinealectomy (PNX + CCl_4_ + MLT) compared to the nontreatment group (PINX + CCl_4_).

**Table 3:** Comparison of Liver Enzyme (AST, ALT, and ALP) Activities

Groups	ALT	AST	ALP
Control	54.37 ± 2.56	139.00 ± 10.63	115.25 ± 6.27
Sham	54.87 ± 2.27	137.87 ± 11.00	149.87 ± 11.07^ h^
PINX	55.75 ± 2.99	140.12 ± 8.53	184.75 ± 11.32^a^
Sham + CCI_4_	298.50 ± 59.93^a,b,c^	382.37 ± 56.12^a,b,c^	295.87 ± 20.71^a,b,c^
PINX + CCl_4_	453.12 ± 63.60^a,b,c,d^	502.50 ± 96.64^a,b,c^	473.25 ± 70.84^a,b,c,d^
PINX + MLT	48.62 ± 1.84^e,f^	106.25 ± 5.09^e,f,h,m^	143.00 ± 10.50^ h,e,f,m^
PINX + CCl_4_ + MLT	222.00 ± 29.72^a,b,c,g,r^	261.25 ± 39.47^ h,k,m,p,s^	145.25 ± 13.87^e,f,m^

Data are expressed as the arithmetic mean ± SE (*n* = 8). AST, aspartate aminotransferase; ALT, alanine aminotransferase; ALP, alkaline phosphatase

^a^ *p* < 0.001 vs Control, ^b^ *p* < 0.001 vs Sham, ^c^ *p* < 0.001 vs PINX, ^d^ *p* < 0.05 vs Sham + CCl_4_, ^e^ *p* < 0.001 vs Sham + CCl_4_, ^f^ *p* < 0.001 vs PINX + CCl_4_, ^g^ *p* < 0.005 vs PINX + CCl_4_, ^h^ *p* < 0.05 vs Control, ^k^ *p* < 0.01 vs Sham, ^m^ *p* < 0.01 vs PINX, ^p^ *p* < 0.05 vs PINX + CCl_4_, ^r^ *p* < 0.001 vs PINX + MLT, ^s^ *p* < 0.005 vs PINX + MLT

### Histopathological Observation in CCl4-Induced Liver Fibrosis Model

**Fig. 3:**
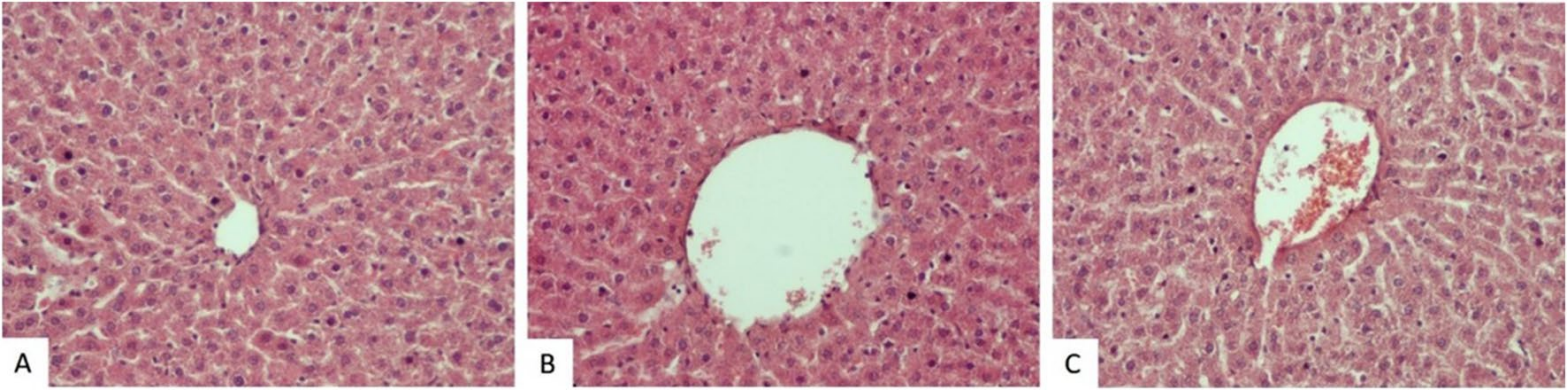
Hematoxylin–eosin staining histological images for the control, sham and PINX groups. Control and sham group liver sections manifested normal histological appearance. PINX group sections were also exhibited near-normal appearance. **A**. Control group, H-E X20. **B**. Sham group, H-E X20. **C**. PINX group, H-E X20.

**Fig. 4:**
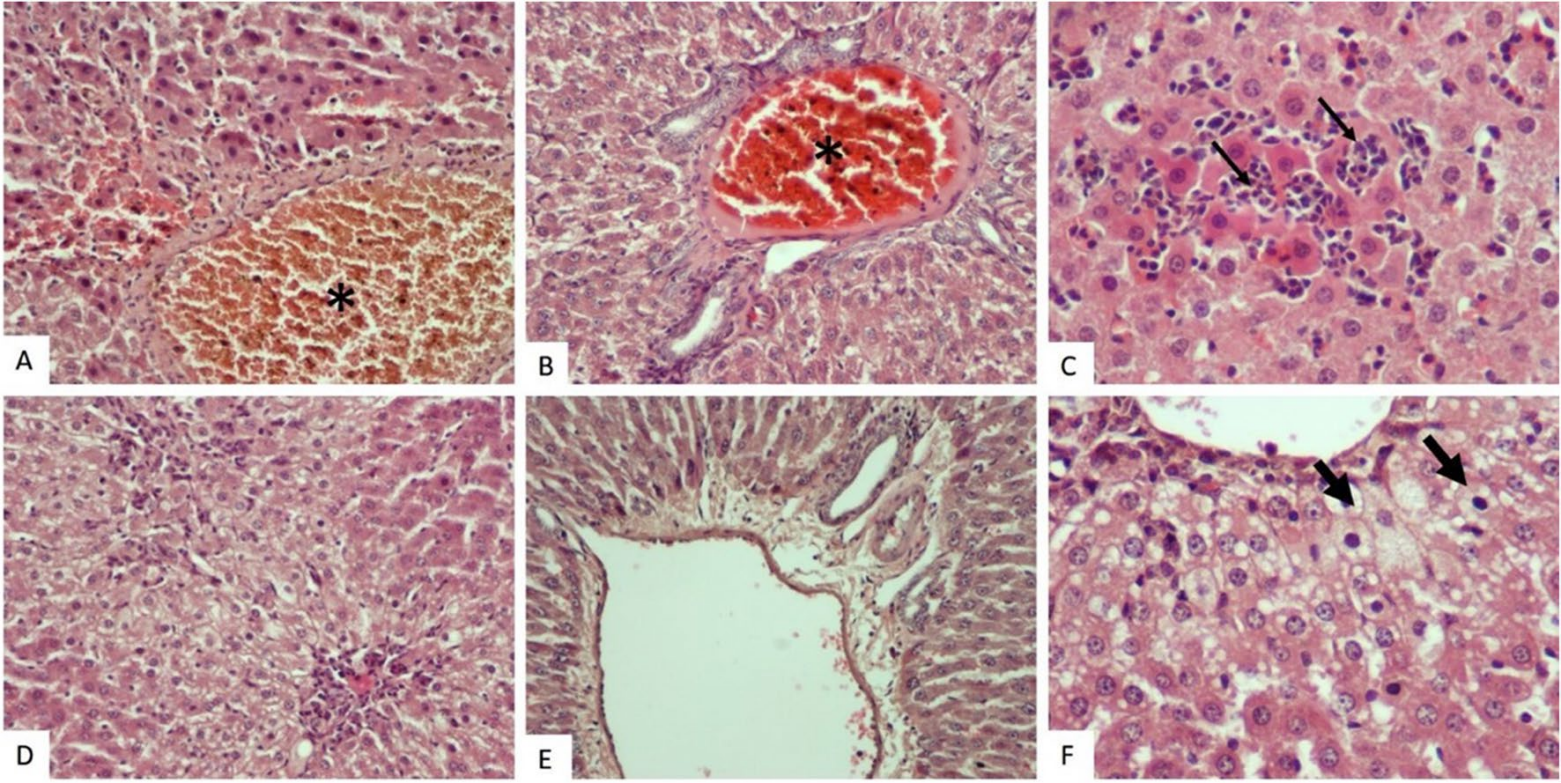
Histological hematoxylin–eosin staining images for the CCl_4_ and PINX + CC_4_ groups. Sham + CCl_4_ and PINX + CCl_4_ groups hepatocyte vacuolization and fatty change (thick arrow), congestion (asterisk), sinusoidal dilatation and inflammatory cell infiltration (thin arrow) and necrosis. **A**. Sham + CCl_4_ group, H-E X20. **B**. Sham + CCl_4_ group, H-E X20. **C**. Sham + CCl_4_ group, H-E X40. **D**. PINX + CCl_4_ group, H-E X20. **E**. PINX + CCl_4_ group, H-E X20. **F**. PINX + CCl_4_ group, H-E X40.

**Fig. 5:**
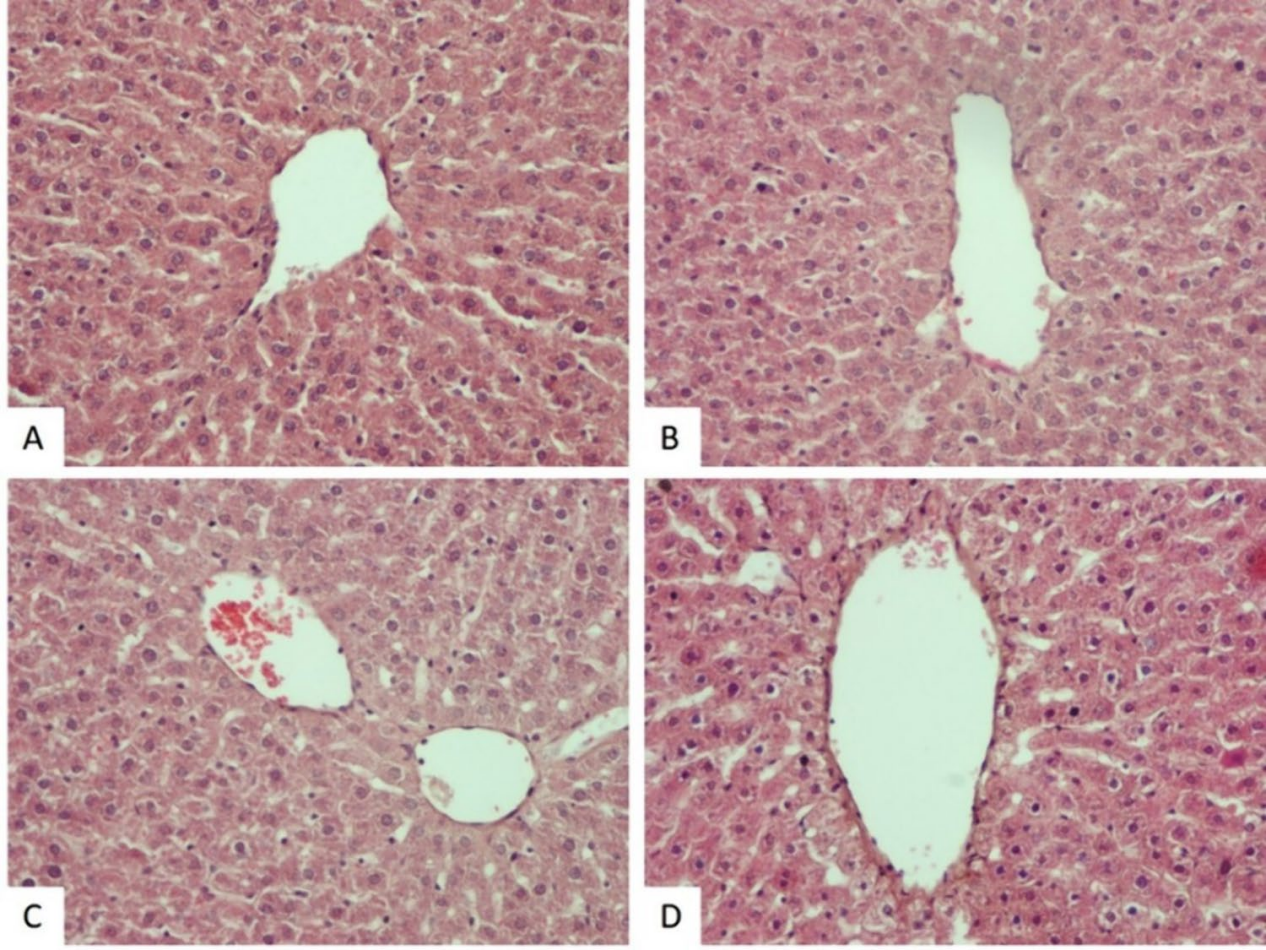
Melatonin administration in PINX + MLT and PINX + CCl_4_ + MLTgroups. The damage was reduced in melatonin administration groups. **A**. PINX + MLT group, H-E X20. **B**. PINX + MLT group, H-E X20. **C**. PINX + CCl_4_ + MLT group, H-E X40. **D**. PINX + CCl_4_ + MLT group, H-E X20.

The histopathological appearance of liver tissue was almost normal in the control (Fig. 3A), sham (Fig. 3B), PINX (Fig. 3C), and PINX + MLT (Fig. 5A-B) groups. However, some changes were observed in H&E-stained liver tissue slides of the Sham + CCl_4_ and PINX + CCl_4_ groups. These changes included vacuolization and fatty deposition, congestion, dilation of sinusoids, and occasional infiltration of inflammatory cells. Some hepatocytes also showed fatty changes, necrosis, and numerous pyknotic cells (Fig. 4). A significant increase in damage scores was observed in the groups treated with CCl_4_ (Sham + CCl_4_ and PINX + CCl_4_ groups) in comparison to the control, Sham, and PINX groups (*p* < 0.001). The mean histopathological damage score (MHDS) was 6.37 ± 0.37 in the Sham + CCl_4_ group and 7.37 ± 0.32 in the PINX + CCl_4_ group (Table 4). On the other hand, melatonin administration improved liver damage caused by CCl_4_ and reduced the damage score in both Sham + CCl_4_ and PINX + CCl_4_ groups (Fig. 5). The MHDS was 2.00 ± 0.37 in the PINX + CCl_4_ + MLT group (Table 4). A statistically significant decrease was observed in the damage scores between the Sham + CCl_4_ and PINX + CCl_4_ groups and the PINX + CCl_4_ + MLT group (*p* < 0.001).

**Table 4:** Mean Histopathological Damage Scores

Groups	Damage Score
Control	0.12 ± 0.12
Sham	0.25 ± 0.16
PINX	0.87 ± 0.22^a^
Sham + CCl_4_	6.37 ± 0.37^b,c,d^
PINX + CCl_4_	7.37 ± 0.32^b,c,d^
PINX + MLT	0.50 ± 0.18^f,g^
PINX + CCl_4_ + MLT	2.00 ± 0.37^b,c,e,f,g,h^

Findings are presented as mean ± standard error (*n* = 8)

^a^ *p* < 0.05 vs Control, ^b^ *p* < 0.001 vs Control, ^c^ *p* < 0.001 vs Sham, ^d^ *p* < 0.001 vs PINX, ^e^ *p* < 0.05 vs PINX, ^f^ *p* < 0.001 vs Sham + CCl4, ^g^ *p* < 0.001 vs PINX + CCl4, ^h^ *p* < 0.005 vs PINX + MLT

### Immunohistochemical Evaluation of TGF-β1 Expression

**Fig. 6:**
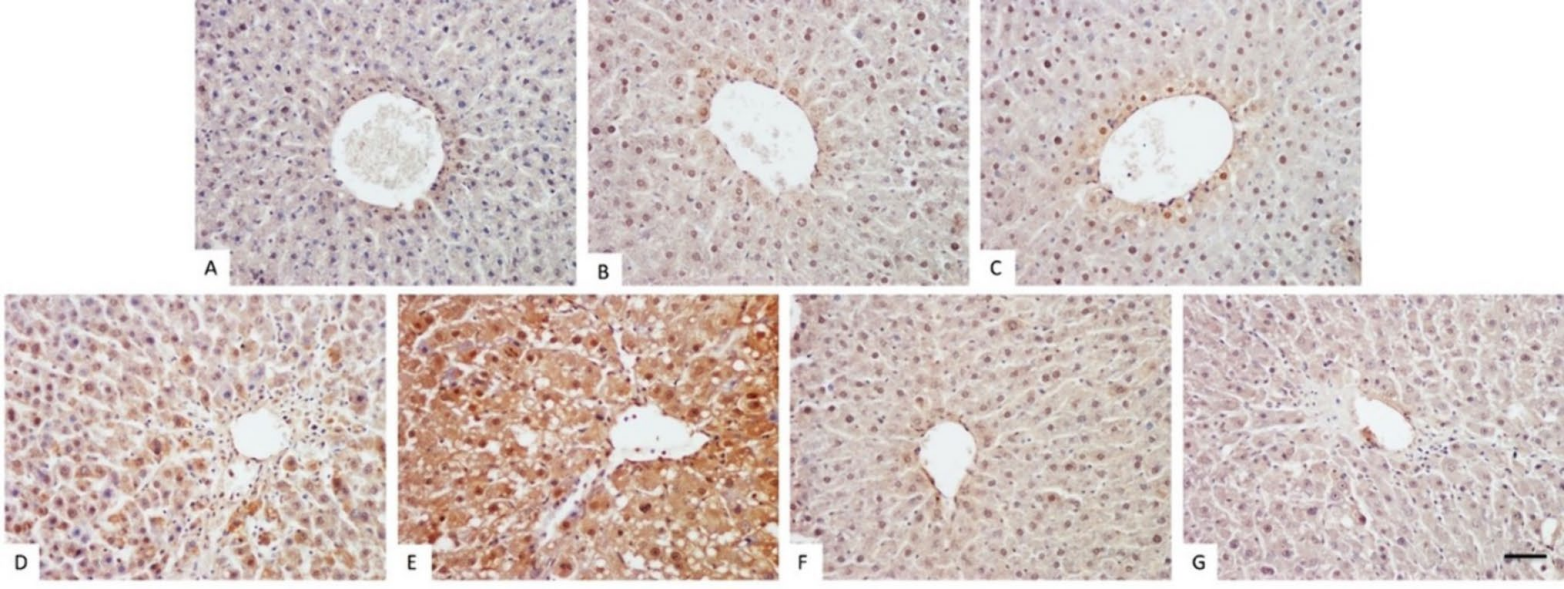
Histological images of the tissue samples stained with anti-TGF B1 antibody with immunohistochemical methods. The tissues were stained with anti-TGF-β1 antibody and the immunohistochemical method. Staining was poor in the control, sham, PINX and PINX + Melatonin groups. The highest expression was observed in the PINX + CCl_4_ group. **A**. Control group. **B**. Sham group. **C**. PINX group. **D**. Sham + CCl_4_ group. **E**. PINX + CCl_4_ group. **F**. PINX + MLT group. **G**. PINX + CCl_4_ + MLT group. Anti-TGF-β1, × 20. Bar: 20 µm.

Fig. 6 revealed that liver tissue slides stained with anti-TGF-β1 antibodies showed higher levels in the Sham+CCl_4_ and PINX+CCl_4_ groups compared to the control, Sham and PINX groups. Furthermore, the PINX+CCl_4_ group exhibited the highest staining levels with anti-TGF-β1 antibody. Administration of melatonin resulted in a reduction of TGF-β1 expression. A significant decrease in expression was observed between the PINX+CCl_4_ and PINX+CCl_4_+MLT groups (*p*<0.001). The H-Score for the TGF-β1 immunostaining is shown in Table 5.

**Table 5:** Mean H-Score for TGF-β1 Immunoreactivity

Groups	H-score
Control	139.25 ± 6.09
Sham	166.25 ± 8.85^a^
PINX	190.00 ± 7.07^b^
Sham + CCl_4_	249.37 ± 7.58^b,c,f^
PINX + CCl_4_	280.62 ± 5.12^b,c,f,h^
PINX + MLT	203.12 ± 1.31^b,d,g,k^
PINX + CCl_4_ + MLT	198.75 ± 8.33^b,e,h,k^

Findings are presented as mean ± standard error (*n* = 8)

^a ^*p* < 0.05 vs Control, ^b ^*p* < 0.001 vs Control, ^c ^*p* < 0.001 vs Sham, ^d ^*p* < 0.05 vs Sham, ^e ^*p* < 0.01 vs Sham, ^f ^*p* < 0.001vs PINX, ^g ^*p* < 0.001 vs Sham + CCl_4_, ^h ^*p* < 0.005 vs Sham + CCl_4_, ^k ^*p* < 0.001 vs PINX + CCl_4_

## DISCUSSION

The potential therapeutic effect of MLT on the CCl_4_-induced fibrotic liver model in pinealectomized rats was investigated in the current study. Our results showed that CCl_4_ administration triggered liver fibrosis through oxidative stress and inflammation in both sham and PINX rats; however, PINX rats were more vulnerable to the toxic effect on the liver tissue of CCl_4_ than Sham rats. Also, it was revealed that MLT supplementation for 15 days could restore liver architecture by regressing CCl_4_-induced liver fibrosis in PINX rats.

The liver plays a vital role in inhibiting toxic substances due to its biochemical and physiological properties. Certain chemicals, such as xenobiotics or their metabolites, could damage the liver. Oxidative stress and free radical production leads to liver damage [30]. CCl_4_ is a chemical agent that causes liver damage due to its hepatotoxic properties. It results in the production of free radicals and tissue damage[31, 32]. Recent reports have verified that CCl_4_ administration could lead to oxidant/antioxidant balance disturbances accompanied by increased ROS, resulting in oxidative stress [33, 34]. Also, the impairment between antioxidants and oxidative indices might cause increased inflammatory mediators, including NF-κB, TNF-α, IL-1 and IL-6 [35, 36]. To prevent oxidative damage, tissues contain enzymatic antioxidant defence systems, such as SOD and CAT, and non-enzymatic antioxidant defence systems, such as GSH and MLT, that have an important role in scavenging oxidants [37].

Oxidative stress is the state where the balance between antioxidants and oxidants favours the oxidants [38]. Oxidative stress-induced ROS formation is quite reactive and oxidizes DNA, lipids, and proteins, causing damage [39]. An increase in oxidative stress leads to the progression of hepatic fibrosis [40]. High oxidative stress plays a key role in the development of liver toxicity [41]. CCl_4_ produces free radicals; thus, it is quite a toxic substance [42]. The hepatotoxic effect of CCl_4_ is based on^.^CCl_3_ and^.^OOCCl_3_ by cytochrome P450 and the hepatic microsomal enzyme [43]. These radicals bind to lipids and cell membrane proteins and stimulate lipid peroxidation, leading to liver damage [44]. Liver damage induced by CCl_4_ entails the steps of reductive dehalogenation, covalent radical binding, inhibition of protein synthesis, fat accumulation, loss in calcium sequestration, apoptosis, and fibrosis [45].

It is known that high oxidative stress is an agent of liver damage [46, 47]. Different doses of CCl_4_ induction with various administration methods would increase lipid peroxidation and reduce GSH levels in liver tissue [48]. One of the methods employed to determine lipid peroxidation is MDA [49]. The MDA levels increase with the increase in oxidative stress [50]. GSH protects the cells from the harmful effects of free radicals. Sun [20] and Chen [43] reported significant decreases in the GSH levels of CCl_4_-induced rats. Kepekçi et al. [51] reported increased MDA levels in liver samples collected 24 h after CCl_4_-induced damages. Previous reports have proven that CCl_4_ administration led to an increase in the levels of ROS, NO, and TBARS in the liver, kidneys, lungs, brain, and spleen of rats while causing a decrease in the levels of enzymatic (SOD and CAT) and non-enzymatic antioxidants (TAS and GSH) [52, 53]. Moreover, several studies have investigated how new therapeutic and protective agents could significantly reduce or prevent CCl_4_-induced tissue toxicities. To our knowledge, no studies have examined the overall therapeutic effects of exogenous MLT administration on the CCl_4_-induced fibrotic liver model in pinealectomized rats and compared the impact of CCl_4_ between normal and pinealectomized rats. Here, the present study evidenced that CCl_4_-induced hepatic fibrosis was revealed by increased oxidative stress, disturbances of the antioxidant system, elevated inflammation, raised TGF-β1 expression and hepatic tissue changes, and these disorders were more severe following CCl_4_ administration after pinealectomy. In fact, consistent with the literature, this study revealed for the first time that CCl_4_ was administered every other day for 15 days in both sham and pinealectomized rats; however, the appearance of hepatic fibrosis upon CCl_4_ administration was more notable in the absence of MLT following the pinealectomy procedure; This was caused by the weakening of the endogenous antioxidant defence system and increased lipid peroxidation in the hepatic tissue.

The SOD enzyme, which dismutates superoxide into H_2_O_2_, plays a role in the initial phase of the antioxidant system. SOD is an important antioxidant that protects aerobic cells from the harmful effects of oxygen radicals. CAT enzyme prevents the accumulation of toxic products after the superoxide dismutase enzyme reaction [54, 55]. High superoxide radical levels inhibit SOD and CAT enzymes. Rat liver toxicity model studies reported significant decreases in SOD and CAT [56, 57]. Consistent with the current research, Raj and Gothandam [58] have reported that CCl_4_ administration causes hepatic injury in Swiss albino mice, leading to a depletion of the antioxidant defence system, including a reduction in SOD, CAT, glutathione S-transferase (GST) and glutathione peroxidase (GPx) and GSH levels in the liver samples. There have been reports that CCl_4_ gives rise to oxidative stress and depletes endogenous antioxidants, including SOD, CAT and GSH [59, 60]. Also, a study by Cheng et al. [61] confirmed that CCl_4_ administration for eight weeks at a dose of 0.4% (1.5 mL/kg body weight,i.p) twice per week was accompanied by a deterioration in liver antioxidant capacity, i.g, a reduction in the SOD, CAT, GPx, and GSH. Moreover, consistent with the various recent studies by Mazani et al. [62] and Wang et al. [63], which reported that CCl_4_-mediated hepatic damage in rats (both 1 mL/kg bw i.p and 20% CCl_4_ orally twice a week) was evidenced by rising lipid peroxidation and collapse in the liver endogenous antioxidant defence system. Consistent with the literature, our results revealed that rats were administered CCl_4_ every other day to sham and PINX rats, resulting in a significant increase in MDA, TOS and OSI levels and a significant decrease in GSH, TAS, SOD and CAT levels. Interestingly, for the first time, the present study evidenced that these changes were more severe in PINX rats than in sham rats following CCl4 administration.

It is known that MLT and its metabolites exhibit antioxidant properties which reduce lipid peroxidation, as well as showing its direct radical scavenging properties and increase the expression of enzymatic antioxidants such as SOD, CAT and GPx [64]. MLT, an amphiphilic compound with an indole ring and methoxy as well as N-acetyl groups, could pass through all lipid bilayers, including the blood–brain barrier, and diffuse simply into all body fluids and cells, leading to elevated power of MLT as an antioxidant [65]. MLT regulates several biological processes, including circadian rhythms, reproduction and sleep [66]. Also, MLT directs cancer cells to apoptosis, inhibits tumour metastasis, suppresses inflammation and serves angiogenesis. This, in turn, the activities of MLT are due to its effects on mitochondria, nuclear receptors, and other intracellular signalling pathways [67]. Interestingly, our results showed that co-administration of CCl_4_ with exogenous MLT after the pinealectomy procedure led to amelioration in the activities of SOD and CAT, elevation in the content of GSH and TAS, and a remarkable decrease in the liver content of MDA and TOS. In the meantime, for the first time, the results of the current study revealed that treatment with exogenous MLT played a crucial role in improving liver antioxidant capacity following CCl_4_ administration in the lack of endogenous MLT, thereby balancing the oxidant/antioxidant state.

CCl_4_ is a potent toxin that produces free radicals (^.^CCl_3_ and^.^OOCCl_3_), which cause damage to the cell membrane and lead to oxidative stress. This stress causes the release of intracellular enzymes, like ALT and AST, which are markers of liver toxicity [68]. High levels of enzymes such as AST, ALT and ALP indicate hepatic dysfunction and damage often seen following CCl_4_ administration [69]. Our experiment has produced compelling evidence that CCl_4_ is severely harmful to the liver. The groups that received CCl_4_ (Sham + CCl_4_ and PINX + CCl_4_) showed a significant increase in ALT, AST, and ALP enzyme levels in comparison to the control groups (Sham and PINX). The increase in these enzyme levels in the plasma was even more pronounced in PINX rats than in Sham rats, which is a novel finding. The current study's findings are consistent with those of Lin et al. [70], who reported that the administration of CCl_4_ to rats for 12 weeks leads to a significant increase in AST, ALT, and ALP levels. The results of Iqbal et al. [71] also support this conclusion, as they found that rats given CCl_4_ three times a week for 28 days at a dose of 2 mL/kg bw experienced a similar increase in these liver enzymes. These findings highlight the importance of CCl_4_ toxicity in inducing liver damage and emphasise the need for further research to explore potential preventive measures. Meanwhile, the administration of Fufang-Liu-Yue-Qing, a traditional Chinese herbal formula, by Lin et al. [70], and Cordia rothii extract by Iqbal et al. [71] in the CCl_4_-induced liver fibrosis model significantly reduced the levels of liver enzymes. Our study confirmed that MLT is one of the important choices due to its potent antioxidant and free radical scavenging properties, which make it an excellent hepatoprotective agent against CCl_4_-induced liver damage. Our latest data showed that MLT treatment to melatonin-deprived rats after administering CCl_4_ could significantly restore cell membrane integrity, reduce plasma ALT, AST, and ALP levels, and enhance the liver's antioxidant status. These benefits are due to MLT's unique ability to scavenge radicals and protect liver cells from oxidative stress. This, in turn, demonstrates the remarkable potential of MLT treatment to promote liver health and function.

Kupffer cells (KCs) are the powerhouse of the liver's monocyte-macrophage system. These cells are responsible for removing harmful bacteria and toxins and play an essential role in anti-inflammatory processes. Moreover, KCs are crucial to chronic inflammatory liver injury as they influence the liver's functions of hepatic stellate cells and endothelial cells [72]. The role of KCs in liver injury, particularly injury induced by CCl_4_, is of utmost importance. In fact, KCs have a crucial role in chemically-induced liver damage models, where they release proinflammatory cytokines such as TNF-α, IL-1, and IL-6 [73]. The release of these cytokines triggers a cascade of toxicity that ultimately damages the liver. These cytokines activate NF-κB, leading to necrosis and apoptosis of liver cells [74]. In addition, stimulating NF-κB could have serious consequences, as it may trigger the release of multiple inflammatory cytokines, including TNF-α and IL-1β [75]. The NF-κB signaling pathway is pivotal in the pathophysiology of numerous inflammatory conditions and is highly susceptible to stimulation by oxidative stress and cytokines [76]. The activation of NF-κB is a crucial step in initiating the transcriptional activation of genes, making it a vital process in biological mechanisms. This process involves sequential phosphorylation of the inhibitor of NF-κB kinase (IKK) and the inhibitor kappa B-α (IκB-α), resulting in incapable of suppressing NF-κB, leading ultimately to the phosphorylation and dimerization of P65. Once the P65 dimer translocates into the nucleus, it binds to response elements in downstream genes, resulting in their transcriptional activation [77]. A study conducted on mice found that administering a dose of 5 μg/g bw of CCl_4_ three times a week for eight weeks led to the activation of the NF-κB signalling pathway. This, in turn, caused a significant increase in the levels of pro-inflammatory cytokines such as TNF-α, IL-1β, and IL-6 [78]. Another study established that CCl_4_ administration at a dose of 2 mL/kg, b.w., i.p twice a week for five weeks in rats led to upregulated NF‐κB, TNF‐α, Cox‐2 and IL‐1β expressions [79]. The current study aligns with previous literature, revealing that CCl_4_ causes hepatic inflammation in rats. However, it is noteworthy for the first time in the current research that the inflammatory effects of CCl_4_ were more profound in pinealectomized rats than in sham rats due to the absence of the modulatory effects of endogenous MLT on the NF-κB signaling pathway. In the present study, the activation of NF-κB and the significant increase of proinflammatory cytokines in rats administered with CCl_4_ led to noteworthy alterations in IL-1β and IL-6 levels. This indicates a strong correlation between the activation of NF-κB and the increase of proinflammatory cytokines, highlighting the importance of controlling these factors to prevent potential health risks. These findings emphasize the importance of MLT and its anti-inflammatory properties in protecting against the harmful effects of CCl_4_-induced inflammation. On the other hand, previous reports have shown that MLT has a protective effect against inflammation by targeting the NF-κB cascade [80–82]. Several studies have demonstrated the effectiveness of MLT supplementation in reducing the expression of NF-κB (p65 subunit), as evidenced by Western blot and immunohistochemical analyses [83, 84]. Furthermore, it has been observed that MLT treatment inhibits the phosphorylation of IκB-α [85]. To examine the anti-inflammatory effects of MLT on CCl_4_-induced hepatic inflammation, we assessed the production of pro-inflammatory cytokines, such as IL-1β and IL-6, as a result of NF-κB activation. This study is the first to demonstrate the modulatory effects of exogenous MLT against CCl_4_-induced hepatic inflammation in pinealectomized rats. Our findings indicate that exogenous MLT treatment reduces inflammation-related parameters in PNX rats, thereby suppressing hepatic inflammation triggered by CCl_4_. Specifically, our study confirms that the protective effects of MLT on hepatic injury induced by CCl_4_ toxicity could be attributed to the suppression of inflammation. In agreement with the present study, Zhang et al.[86] reported that MLT regulated the expression of TNF-α and IL-6 in CCl_4_-induced liver injury.

The liver's extracellular matrix (ECM) is continually synthesized and degraded in a balanced manner. However, once the liver is injured, this balance is disrupted, activating matrix metalloproteinases (MMPs) associated with fibrosis. Fibrosis is a process that involves dynamic changes in the ECM. During this process, certain endogenous factors such as interleukin-17 (IL-17) are released, which then stimulate KCs to produce other pro-inflammatory factors like TGF-β1 and TNF-α [87]. TGF-β1 also stimulates hepatic stellate cells (HSC) and activates collagen expression, causing an increase in ECM deposition. Thus, inhibiting TGF-β1 is crucial for effectively preventing liver fibrosis [88]. Normally, bile acids produced in hepatocytes are excreted into the bile duct, then collected in the common bile duct and emptied into the duodenum, with only a small amount of bilirubin and bile acid present in the blood (1). Under pathological conditions, blocked bile flow leads to the accumulation of bile acids and bound bile salts in hepatocytes and serum. Higher levels of bile acids, such as glycochenodeoxycholate (GCDC), are more toxic and can cause apoptosis by interfering with mitochondrial function or directly leading to cell death [89]. If the obstructive factors are not resolved in time, it can eventually lead to liver fibrosis and cirrhosis [90]. To analyze the anti-fibrotic effects of MLT on CCl_4_-induced liver fibrosis, we conducted an immunohistochemical observation to determine the increased TGF-β1 expression resulting from KCs activation. Our findings showed that TGF-β1 was elevated in both sham and PINX rats following CCl_4_ administration. Furthermore, we observed, for the first time, that the expression of TGF-β1 was significantly higher in PINX rats than in Sham rats after CCl_4_ administration. To our knowledge, this is the first study to investigate the inhibitory effects of exogenous MLT on CCl4-induced hepatic fibrosis in rats that underwent pinealectomy. Herein, the current study revealed that exogenous MLT treatment restored liver fibrosis compared to the non-MLT treated group through inhibition of TGF-β1. The results of the present study agree with the findings of Zhang et al. [86], who reported a significant decrease in MMP-9, TGF-β1, and MMP-2-positive cells following MLT treatment in CCl_4_-administered rats. Also, Wang et al. [91] showed that MLT suppressed liver fibrosis caused by CCl_4_ by inhibiting TGF-β1/Smad pathway. This result was confirmed by the decreased expression of TGF-β1, Smad2/3, and p-Smad2/3 and the increased expression of Smad7 in the liver. These findings strongly suggest that MLT could be a promising therapeutic agent for counteracting the harmful effects of CCl_4_-induced toxicity.

Histopathologically, the current study confirmed that CCl_4_ caused significant histological changes in the liver. These changes include vacuolization and fatty deposition, congestion, dilation of sinusoids, and occasional infiltration of inflammatory cells in the hepatocellular cells. Furthermore, the study found that sham and PINX rats showed fatty changes, necrosis, and numerous pyknotic cells in some hepatocytes after CCl_4_ administration. However, exogenous MLT treatment restored the architecture of the liver in CCl_4_-induced liver fibrosis. Consistent with the current study, Zhang et al. [86] and Wang et al. [91] demonstrated that MLT treatment led to a significant improvement in liver fibrosis following CCl_4_ administration, as confirmed by histopathological evaluation.

In conclusion, the present study has demonstrated that exogenous MLT offers a promising solution to ameliorate the CCl_4_-induced liver fibrosis model even without physiological MLT. The protective effects of MLT are primarily attributed to its potent antioxidant and anti-inflammatory properties, as well as its ability to inhibit TGF-β1 expression on injured hepatocytes. However, further research is required to thoroughly investigate its impact on oxidative stress, inflammatory response, and TGF-β1 expression and its correlation with endogenous MLT following CCl_4_-induced liver fibrosis to fully comprehend the mechanical actions of exogenous MLT.

## Data Availability

No datasets were generated or analysed during the current study.
